# Stimulation of a protease targeting the LRIM1/APL1C complex reveals specificity in complement-like pathway activation in *Anopheles gambiae*

**DOI:** 10.1371/journal.pone.0214753

**Published:** 2019-04-08

**Authors:** Valeria M. Reyes Ruiz, Gregory L. Sousa, Sarah D. Sneed, Katie V. Farrant, George K. Christophides, Michael Povelones

**Affiliations:** 1 Department of Microbiology, Perelman School of Medicine, University of Pennsylvania, Philadelphia, Pennsylvania, United States of America; 2 Department of Pathobiology, School of Veterinary Medicine, University of Pennsylvania, Philadelphia, Pennsylvania, United States of America; 3 Department of Life Sciences, Imperial College London, London, United Kingdom; National Institutes of Health, UNITED STATES

## Abstract

The complement-like pathway of the African malaria mosquito *Anopheles gambiae* provides protection against infection by diverse pathogens. A functional requirement for a core set of proteins during infections by rodent and human malaria parasites, bacteria, and fungi suggests a similar mechanism operates against different pathogens. However, the extent to which the molecular mechanisms are conserved is unknown. In this study we probed the biochemical responses of complement-like pathway to challenge by the Gram-positive bacterium *Staphyloccocus aureus*. Western blot analysis of the hemolymph revealed that *S*. *aureus* challenge activates a TEP1 convertase-like activity and promotes the depletion of the protein SPCLIP1. *S*. *aureus* challenge did not lead to an apparent change in the abundance of the LRIM1/APL1C complex compared to challenge by the Gram-negative bacterium, *Escherichia coli*. Following up on this observation using a panel of LRIM1 and APL1C antibodies, we found that *E*. *coli* challenge, but not *S*. *aureus*, specifically activates a protease that cleaves the C-terminus of APL1C. Inhibitor studies in vivo and in vitro protease assays suggest that a serine protease is responsible for APL1C cleavage. This study reveals that despite different challenges converging on activation of a TEP1 convertase-like activity, the mosquito complement-like pathway also includes pathogen-specific reactions.

## Introduction

Mosquitoes are a global disease threat as they transmit numerous human and animal pathogens. Mosquitoes have a powerful innate immune system that protects them from infections by blood-acquired pathogens as well as those encountered in their environment. The most devastating mosquito-borne disease is malaria, which killed 435,000 individuals in 2017 [1]. *Anopheles gambiae* is the major vector of human malaria in sub-Saharan Africa, and there is therefore considerable interest in understanding how its immune system responds to *Plasmodium* parasites and other microbes. Motile *Plasmodium* ookinetes, a stage formed in the blood bolus upon parasite sexual reproduction, traverse mosquito midgut epithelial cells and contact the hemolymph-filled body cavity (hemocoel) [2–4]. Ookinete exposure to the hemolymph drives activation of the complement-like pathway, a potent immune reaction that results in a dramatic reduction in viable parasites, thus constituting a major immune barrier that robustly limits infection by human and rodent malaria parasites [5, 6].

A key event in mosquito complement activation is the accumulation of a thioester-containing protein, TEP1, on the surface of ookinetes [7]. TEP1 structurally and functionally resembles the C3 component of the vertebrate complement system [8], possessing a highly reactive thioester motif that allows it to make covalent linkages to molecules on the pathogen surface [9]. TEP1 is constitutively expressed and present in the hemolymph as both a 150 kDa full-length protein (TEP1-F) [9], in which the reactive thioester is buried in a hydrophobic pocket [8], and a processed form (TEP1_cut_), where the thioester is stabilized by an interaction with a disulfide-linked heterodimer of two Leucine-rich repeat (LRR) Immune Proteins, LRIM1 and APL1C [10, 11]. During mosquito complement activation, TEP1-F is processed to TEP1_cut_ and it is delivered to microbial surfaces. This is a convertase-like reaction that requires the non-catalytically active CLIP-domain serine protease homolog SPCLIP1 [7, 12, 13]. Accumulation of TEP1 promotes lysis and, in some contexts, melanization of *Plasmodium* ookinetes in a mechanism that requires another serine protein homolog, CLIPA8 [7, 14].

Like vertebrate C3, TEP1 is central to defense against different pathogens. For example, in addition to *Plasmodium* defense, the *An*. *gambiae* complement pathway protects the mosquito against bacterial and fungal infections [15–17]. Silencing TEP1 leads to a strong reduction in phagocytosis of *Escherichia coli* and *Staphyloccocus aureus* [9, 16]. TEP1 silencing also significantly reduces survival to challenges with *E*. *coli*, *S*. *aureus*, and *Beauvaria bassiana*, an entomopathogenic fungi [13, 15]. Interestingly, silencing other components of the complement pathway produces infection-specific phenotypes. For example, silencing LRIM1 dramatically inhibits phagocytosis of *E*. *coli*, but does not affect *S*. *aureus* [16]. Furthermore, different effector functions downstream of TEP1 appear to be specialized to neutralize different pathogens. For example, melanization is not required for antibacterial defense, but it does play an important role in antifungal defense [13, 18]. Melanization of *Plasmodium* ookinetes is also observed in different refractory mosquito models and is associated with enhanced parasite killing [7, 17, 19, 20]. This suggests that although TEP1 is universally required for defense against diverse infections, other components may be pathogen-specific.

Previous studies utilized the Gram-negative bacterium, *E*. *coli*, as a model to dissect the molecular mechanisms of the mosquito complement response relevant to *Plasmodium* infection [12]. TEP1-F is strongly utilized during *E*. *coli* challenge by activation of a convertase-like activity in a mechanism requiring SPCLIP1. In addition, the LRIM1/APL1C heterodimer is shown to decrease in abundance in the hemolymph following *E*. *coli* challenge, suggesting that it is localized to the microbial surface [12]. Here, we look at the molecular events following challenge with a Gram-positive bacterium, *S*. *aureus*, to address the similarities and aforementioned differences between the mechanisms that control *E*. *coli* and *S*. *aureus* challenge using a combination of gene silencing and biochemical analyses. Our data demonstrate, for the first time, the molecular and functional specificity for the mosquito complement pathway in response to diverse microbial challenge.

## Results

### *S*. *aureus* challenge promotes complement activation and utilization of TEP1-F

**Fig 1:**
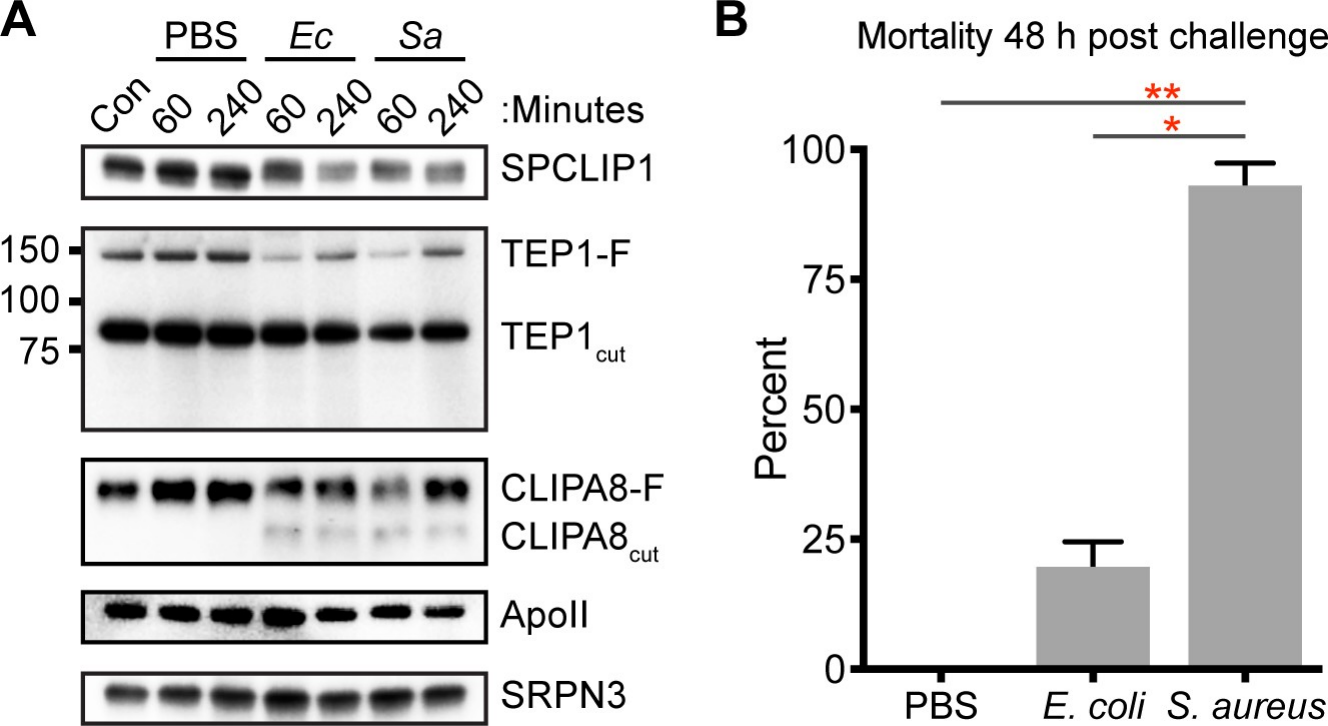
*E*. *coli* and *S*. *aureus* bioparticles challenge trigger similar complement-like pathway responses but differences in survival. (**A**) Western blot analysis of SPCLIP1, TEP1 and CLIPA8 in mosquito hemolymph collected from control (Con) and after injection of PBS, or chemically killed *E*. *coli* (*Ec*) or *S*. *aureus* (*Sa*) bioparticles. Blots were probed with SRPN3 and Apolipophorin II/I (ApoII) antibodies to confirm equal loading. Labels on the right indicate protein or proteolytic products detected. Markers on the left of the TEP1 panel indicate molecular weight in kDa. Images are representative of three independent biological replicates performed with both *An*. *gambiae* N’gousso strain mosquitoes. (**B**) Mean percent mortality 48 hours after injection with PBS, *E*. *coli* bioparticles or *S*. *aureus* bioparticles. Data presented are the average of three independent generations of *An*. *gambiae* G3 strain mosquitoes. The error bars indicate the standard error of the mean. As indicated, the *S*. *aureus* bioparticles challenged group is significantly different than *E*. *coli* bioparticles and PBS challenges. Challenge with *E*. *coli* is not significantly different from the control (P = 0.102). Asterisks indicates ANOVA P-value < 0.05 (*) and < 0.005 (**) with correction for multiple comparisons.

To compare how the complement-like pathway responds to distinct microbial surfaces, *An*. *gambiae* hemolymph was biochemically analyzed after challenge with *E*. *coli* or *S*. *aureus*. We took advantage of a challenge model we previously established utilizing killed bacteria (bioparticles) to prevent any confounding effects due to bacterial proliferation [12]. The number of bacteria injected was constant to compare the responses to these different cell types. Hemolymph was collected at 60 and 240 minutes after injection of bacteria into the mosquito hemocoel. Western blot analysis revealed that injection of *E*. *coli* and *S*. *aureus* bioparticles results in decreased SPCLIP1 from the hemolymph and a concomitant depletion of TEP1-F at both time points compared to untreated or buffer-injected control groups (Fig 1A). TEP1-F levels are higher at 240 minutes compared to 60 minutes indicating that its rate of consumption is lower than its synthesis at this time point. Both *E*. *coli* and *S*. *aureus* challenge led to the rapid and sustained cleavage of CLIPA8 indicated by presence of a faster migrating form. These observations indicate that Gram-positive *S*. *aureus* surfaces trigger the formation of a TEP1 convertase and the cleavage of CLIPA8, potentially resulting in downstream effector functions, such as the melanization cascade, similar to what was previously reported for Gram-negative *E*. *coli* surfaces [12]. Concomitant with the depletion of SPCLIP1 following bacterial challenge we observe the band as a dimer. Whether this is the result of a necessary activation cleavage for SPCLIP1 remains to be determined. Though we did not observe any differences in mosquito mortality at 240 minutes post challenge, we found that *S*. *aureus* challenge resulted in significantly higher mosquito mortality 48 hours post injection compared to either the *E*. *coli* injected group or the PBS injected controls (Fig 1B). Mortality was extensive and ranged from 85–100% of the *S*. *aureus*-treated mosquitoes. Challenge with *E*. *coli* had a milder increase in mortality (ranging from 11–28%) that was not significant compared to the control. The differences in mortality induced by *S*. *aureus* and *E*. *coli* treatment are interesting given the similarities we observed with the complement protein responses.

### SPCLIP1 is required for CLIPA8 cleavage but not TEP1 utilization in response to *S*. *aureus* challenge

**Fig 2:**
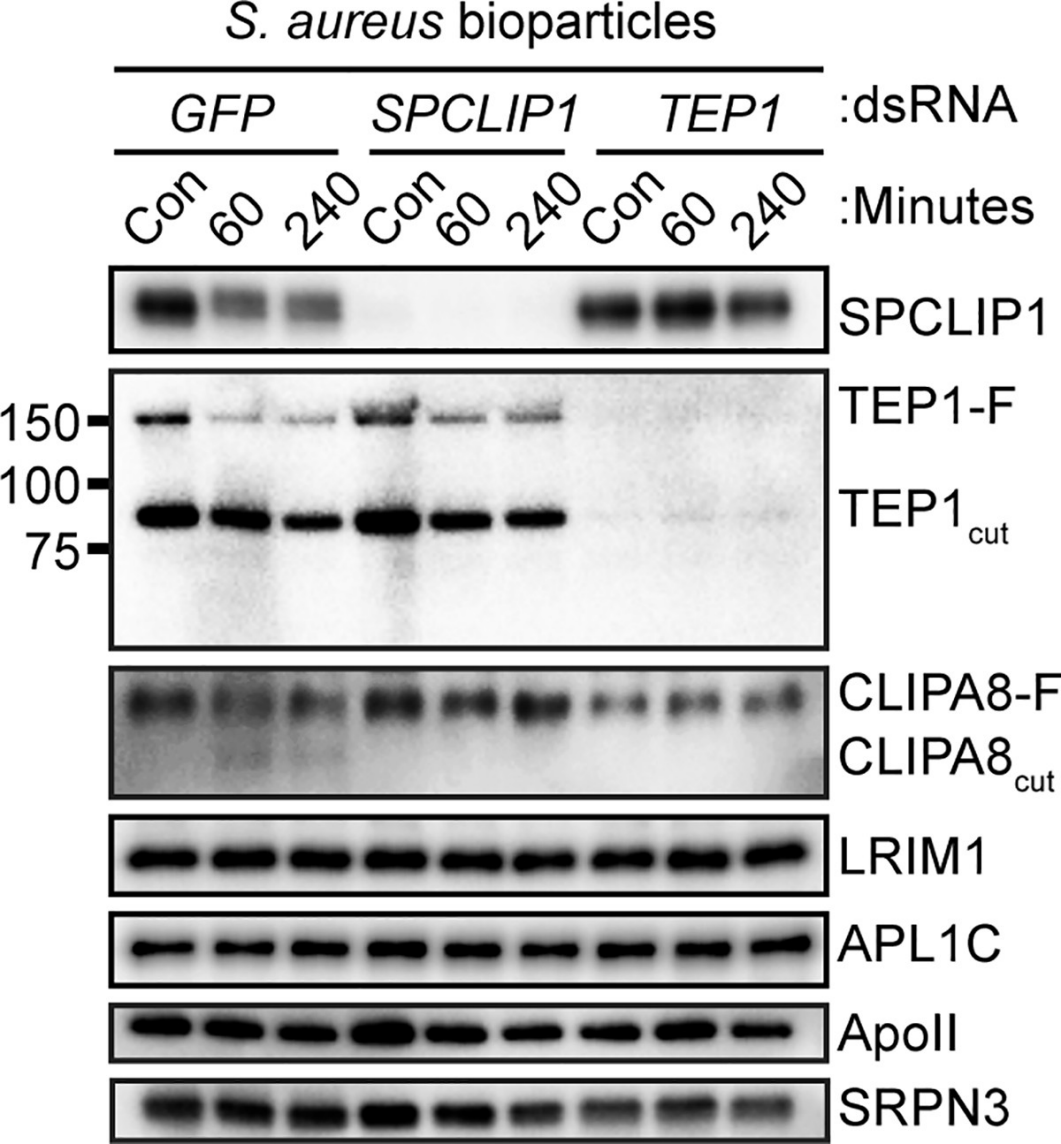
SPCLIP1 and TEP1 are required for CLIPA8 cleavage following *S*. *aureus* challenge. Western blot analysis of hemolymph collected from ds*GFP*-injected, *SPCLIP1* kd, and *TEP1* kd mosquitoes following no treatment (Con) or injection with *S*. *aureus* bioparticles. Blots were probed for ApoII and SRPN3 to confirm equal loading. Labels on the right indicate protein or proteolytic products detected. Images are representative of three independent biological replicates using *An*. *gambiae* N’gousso strain mosquitoes.

SPCLIP1 is required for the recruitment of TEP1 to the surface of *E*. *coli* and *P*. *berghei* during infection [12]. To test whether the loss of TEP1-F observed following infection with *S*. *aureus* requires SPCLIP1, we challenged mosquitoes after knockdown of SPCLIP1. Following treatment with *SPCLIP1* dsRNA there was a strong depletion of the SPCLIP1 protein (Fig 2). Despite this loss, TEP1-F utilization was not dramatically affected during *S*. *aureus* challenge compared to ds*GFP*-treated controls. In both treatments, there was still a robust loss of TEP1-F. In contrast, the knockdown of SPCLIP1 completely prevented the cleavage of CLIPA8.

We next determined whether the *S*. *aureus*-induced depletion of SPCLIP1 and cleavage of CLIPA8 requires TEP1. Mosquitoes treated with *TEP1* dsRNA showed very efficient knockdown, as both TEP1-F and TEP1_cut_ were virtually undetectable by western blot compared to ds*GFP*-injected controls (Fig 2). We found that TEP1 silencing prevented the loss of SPCLIP1 following *S*. *aureus* challenge (Fig 2). Similar to untreated controls (Fig 1), the ds*GFP* treated control group showed decreased SPCLIP1 at 60 and 240 minutes following injection of killed *S*. *aureus*, whereas after TEP1 knockdown, SPCLIP1 levels in the hemolymph remained comparable to unchallenged controls. These results suggest that depletion of SPCLIP1 following *S*. *aureus* challenge requires TEP1 (Fig 2). TEP1 knockdown also completely prevented the CLIPA8 cleavage observed in the ds*GFP*-treated controls (Fig 2). The effects on SPCLIP1 and CLIPA8 were specific as there were no apparent differences in the abundance of complement pathway components LRIM1 or APLIC or in two loading controls, ApoII [21] and SRPN3 [22] (Fig 2).

### *E*. *coli* challenge promotes the cleavage of APL1C

**Fig 3:**
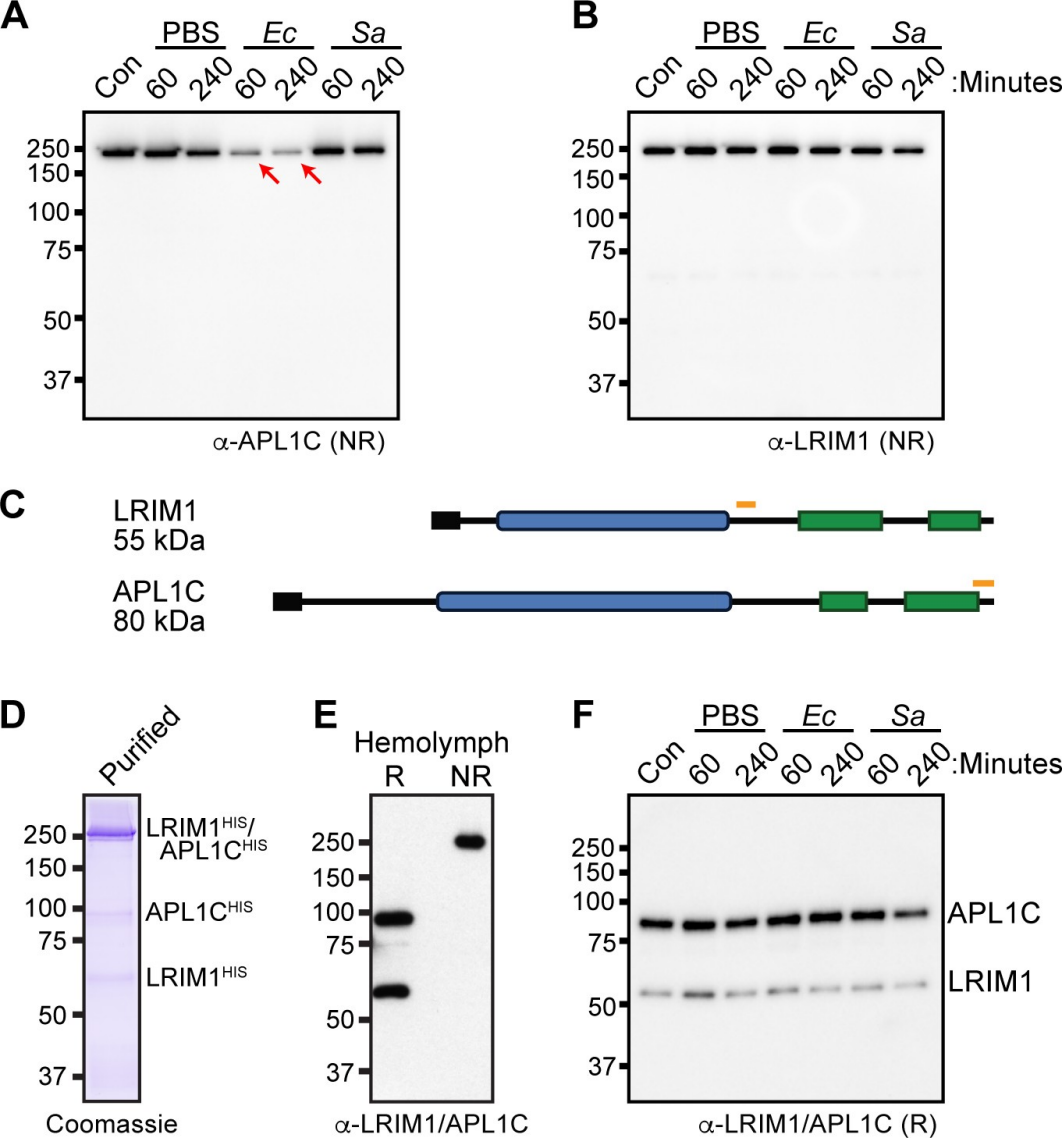
*E*. *coli* surfaces trigger the loss of specific C-terminal epitope of APL1C. Western analysis of hemolymph collected from control (Con) and after injection of PBS, *E*. *coli* (*Ec*), or *S*. *aureus* (*Sa*) bioparticles. (**A**) The LRIM1/APL1C complex was detected under non-reducing (NR) conditions using an anti-peptide antibody directed against the C-terminus of APL1C. Red arrows indicate the loss of signal following *E*. *coli* injection. (**B**) The LRIM1/APL1C complex was detected under NR conditions using an anti-peptide LRIM1 antibody directed against an internal epitope. (**C**) Cartoon of the LRIM1/APL1C complex with the approximate locations of the peptides used to generate LRIM1 and APL1C specific antibodies indicated by an orange box. The N- and C-termini and predicted molecular weight of each protein indicated in gray. Adapted from (41). (**D**) His-tagged LRIM1/APL1C heterodimer and monomers affinity purified from the conditioned medium of Sf9 cells expressing LRIM1^HIS^ and APL1C^HIS^. These proteins were used as antigens to produce polyclonal antiserum. (**E**). Western blot analysis of hemolymph under reducing (R) and NR conditions using LRIM1/APL1C polyclonal antibody. **(F)** Western blot analysis of LRIM1 and APL1C under R conditions using the LRIM1/APL1C polyclonal following PBS, *Ec*, and *Sa* challenge. Images are representative of two independent biological replicates using *An*. *gambiae* N’gousso strain mosquitoes.

We previously reported that the LRIM1/APL1C complex was dramatically reduced in the hemolymph after injection of killed *E*. *coli*, prompting the hypothesis that it bound to the bacterial surface and was required for recruitment of TEP1-F [12]. We wanted to determine whether LRIM1/APL1C is also depleted from the hemolymph following *S*. *aureus* challenge. We assayed the LRIM1/APL1C heterodimer in hemolymph in response to challenge with killed *S*. *aureus* and *E*. *coli* using an antibody against APL1C. Strikingly, we found that although *E*. *coli* resulted in a strong reduction in the intensity of the band detected, the signal strength following *S*. *aureus* treatment was unaffected (Fig 3A), indicating specificity in the mechanism of complement-like pathway activation.

We next analyzed the same samples using an antibody raised against LRIM1 we previously characterized (Fig 3B) [11]. Given our hypothesis that *E*. *coli* promoted the depletion of the entire LRIM1/APL1C complex, it was therefore unexpected to find that neither *E*. *coli* nor *S*. *aureus* injection changed the abundance of the LRIM1/APL1C heterodimer as detected by the LRIM1 antibody. The antigenic peptides used to produce these antibodies are shown in Fig 3C. Given that the APL1C antibody used for this assay is directed against the C-terminal tail of APL1C, and that the abundance and apparent size of the LRIM1/APL1C complex remained constant in both challenges when assayed with the LRIM1 antibody, we hypothesized that the apparent *E*. *coli*-specific depletion of the complex observed when using the APL1C antibody was due to proteolytic cleavage of the APL1C tail of the circulating LRIM1/APL1C complex and not due to the localization of the heterodimer to the bacterial surface.

To directly test this hypothesis, we generated a new polyclonal antibody against APL1C to examine whether APL1C is still present in the hemolymph following bacterial challenge. This antibody was generated by immunizing guinea pigs with full-length LRIM1 and APL1C affinity purified from conditioned medium of Sf9 cells co-expressing His-tagged LRIM1 and APL1C (Fig 3D). The conditioned medium contains LRIM1^HIS^/APL1C^HIS^ heterodimer, as well as LRIM1^HIS^ and APL1C^HIS^ homodimers and monomers. Western blotting of hemolymph run under non-reducing conditions showed that the antiserum recognizes a single prominent band migrating at the size of the LRIM1/APL1C heterodimer in mosquito hemolymph (Fig 3E). In a reduced sample, the heterodimer dissociates revealing that the antiserum contains antibodies against both LRIM1 and APL1C and recognizes both proteins with similar efficiency. When we probed our previous hemolymph samples using the new LRIM1/APL1C polyclonal antiserum under reducing conditions, we found that both APL1C and LRIM1 were maintained in the hemolymph in all conditions (Fig 3F). These data demonstrate that the loss of signal observed with the APL1C peptide antibody following *E*. *coli* challenge is indeed due to proteolysis or destruction of the epitope and not deposition of the LRIM1/APL1C complex on the microbial surface. We note that proteolysis must be limited since there were no observable changes in mobility of the LRIM1/APL1C complex (Fig 3B) or in the APL1C protein (Fig 3F) even when samples were analyzed using different single percentage gels or using gradient gels.

### Protease inhibitor treatment reduces APL1C cleavage following *E*. *coli* challenge

**Fig 4:**
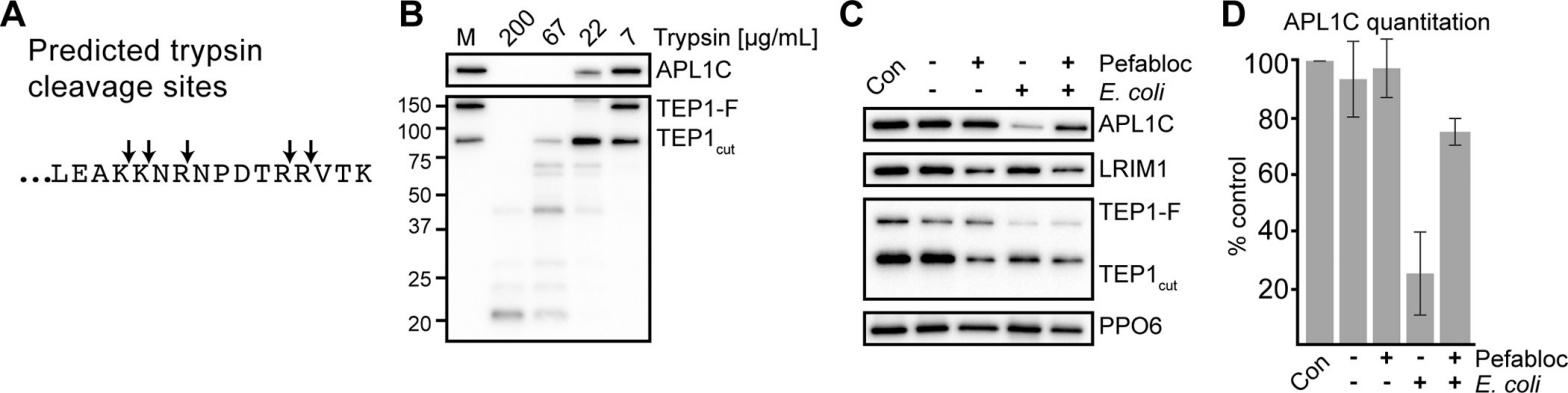
The C-terminus of APL1C is processed by serine protease specifically after *E*. *coli* challenge. (**A**) Arrows indicate the position of five predicted trypsin cleavage sites in the C-terminal 16 amino acids of the APL1C protein. (**B**) Non-reducing western blot analysis of TEP1 and the LRIM1/APL1C complex, detected with an APL1C anti-peptide antibody, in Sua4.0 conditioned medium treated for 30 minutes with different concentrations of porcine trypsin or mock-treated (M) (**C**) Non-reducing western analysis of hemolymph collected from control (Con) and 15 minutes after injection of *E*. *coli* bioparticles (*Ec*). The LRIM1/APL1C complex was detected with both APL1C and LRIM1 anti-peptide antibodies. Immediately before the bacterial injection, mosquitoes were injected with water or the serine protease inhibitor Pefabloc. **(D)** Two independent experiments shown in panel C were used to quantitate the APL1C band. The average level of APL1C compared to control is shown following normalization to the loading control PPO6. Error bars indicate the standard deviation. Images in this figure are representative of two independent biological replicates using *An*. *gambiae* G3 strain mosquitoes.

To examine the hypothesis that *E*. *coli* induces a limited proteolysis of the APL1C tail, we analyzed the C-terminal tail of APL1C bioinformatically for predicted protease cleavage sites using the ExPASy PeptideCutter [23]. We found that the last 16 amino acids used for the generation of the anti-peptide antibody (Fig 3C) [11] have 5 predicted cleavage sites for trypsin, a serine-protease (Fig 4A). Given these predicted trypsin cleavage sites, we tested whether purified trypsin could promote the cleavage of the APL1C. Trypsin was added in different concentrations to conditioned medium prepared from *An*. *gambiae* cultured cells known to secrete the LRIM1/APL1C complex, TEP1-F and TEP1_cut_ [11]. Following a limited proteolysis, the conditioned medium was analysed by non-reducing SDS-PAGE. Incubation with 22 μg/mL trypsin caused a decrease in APL1C signal resembling what we observed in mosquito hemolymph following *E*. *coli* challenge (Fig 4B). In contrast to what we observed in the hemolymph, this treatment also led slight increase in the mobility of the LRIM1/APL1C complex. As expected, based on previous work, this concentration of trypsin also promoted a conversion of TEP1-F into TEP1_cut_ along with three minor smaller fragments [8]. Higher concentrations of trypsin completely abrogate APL1C signal and lead to the further fragmentation of TEP1. Lower concentrations had no effect on either APL1C or TEP1. To test whether a serine protease is responsible for the in vivo cleavage of APL1C, we repeated the *E*. *coli* challenge in the presence of Pefabloc, a water-soluble irreversible serine protease inhibitor. First, we injected mosquitoes with Pefabloc or water alone. After a 15-minute incubation, mosquitoes were injected with killed *E*. *coli* in PBS or with PBS alone. Non-reducing western blot analysis of the LRIM1/APL1C complex from hemolymph collected 60 minutes after challenge using the APL1C peptide antibody showed that Pefabloc pre-treatment blocked the reduction of the APL1C signal stimulated by *E*. *coli* challenge (Fig 4C). We quantitated the effect and found that there was only a 20% reduction in APL1C signal following *E*. *coli* challenge in the presence of Pefabloc compared to challenge in its absence, which resulted in an 80% reduction (Fig 4D). In contrast, the levels of the LRIM1/APL1C complex detected with the LRIM1 antibody or the loading control, PPO6, were not affected by *E*. *coli* challenge or Pefabloc treatment (Fig 4C). These results suggest that a serine protease is required for the *E*. *coli*-specific cleavage of the APL1C tail. Given that this assay was performed *in vivo*, it is unknown whether Pefabloc inhibits a serine protease that acts directly on the APL1C tail or on a required upstream protease. Interestingly, in these samples we did not observe a block in the utilization of TEP1-F following *E*. *coli* challenge. This suggests that although TEP1-F can be processed to TEP1_cut_ by a serine protease *in vitro* (Fig 4B) [8], it may be processed *in vivo* by a class of protease that is insensitive to Pefabloc. Alternatively, the inhibitor concentration used in this experiment is not sufficient to prevent the conversion of TEP1-F to TEP1_cut_.

## Discussion

Insects have a powerful innate immune system that can launch distinct pathogen-specific responses. Triggering different pathways allows pathogen-appropriate effector responses. Work in *Drosophila* has elucidated mechanisms for differential sensing of Gram-negative and Gram-positive bacteria through specific activation of the Imd and Toll pathways, respectively [24]. The mosquito, *An*. *gambiae* relies on a complement-like pathway to defend against diverse infections of the hemolymph. To date, the same components and molecular mechanisms have been shown to mediate the mosquito complement response to different infections. Here we provide the first evidence of infection-specific molecular events during mosquito complement activation.

Specificity in vertebrate complement is achieved through interactions between either the Mannose-Binding Lectin (MBL) complex and pathogen-associated carbohydrates or between Complement component 1q (C1q) and pathogen-specific immunoglobulins [25]. In each case, specific zymogen proteases associated with the MBL or C1q complex are activated by conformational changes induced by pathogen binding. Despite proceeding via different initial events, both MBL and C1q binding ultimately converge on the generation of a C3 convertase required for C3 utilization and activation of effector functions [25]. In mosquitoes, it seems clear that if there are distinct modes of activation for the complement-like pathway that they converge on TEP1, a C3-like molecule.

**Fig 5:**
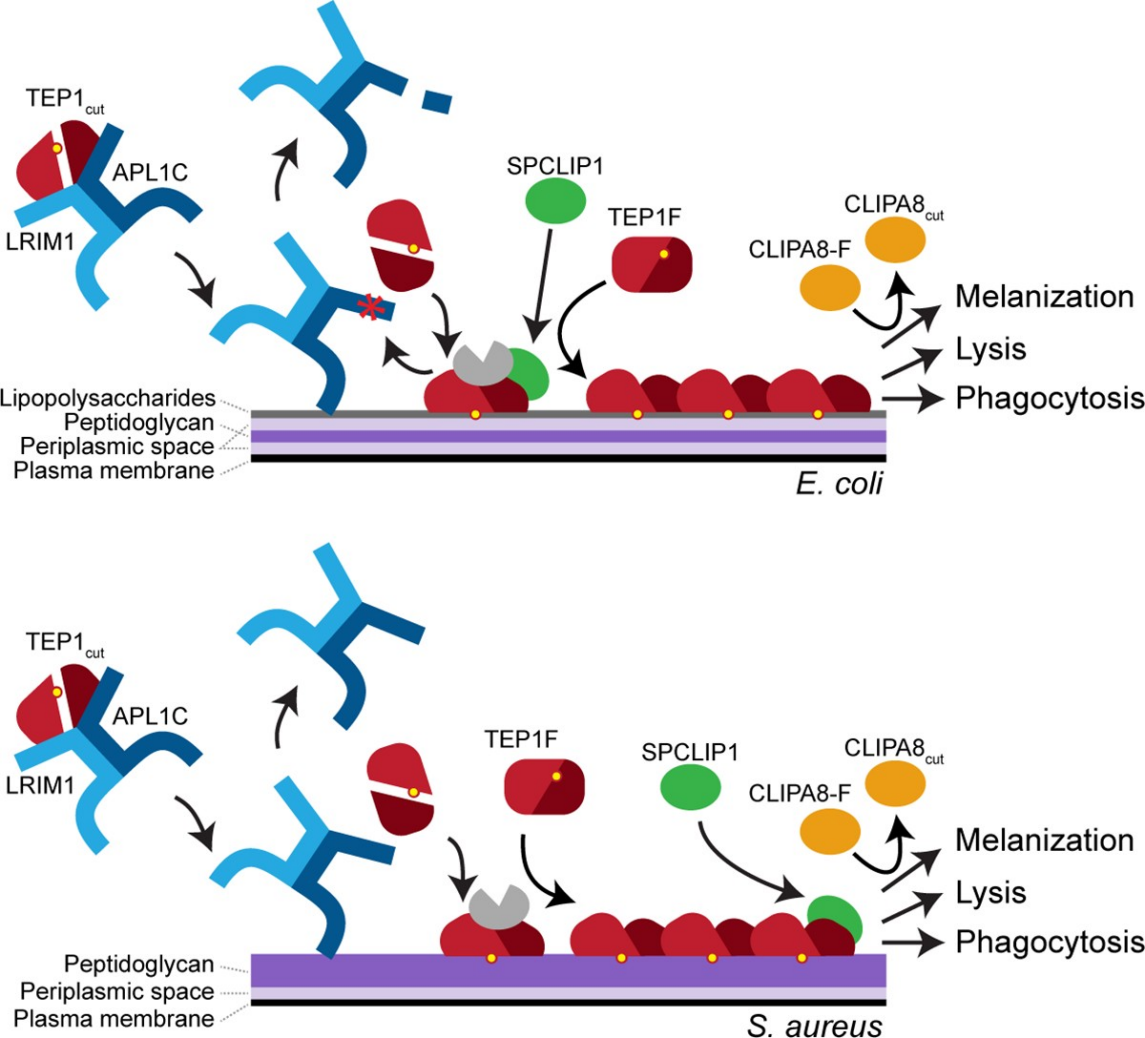
Model of complement-like pathway activation following *E*. *coli* and *S*. *aureus* challenge. *E*. *coli* and *S*. *aureus* differ in the composition of their cell surface. *E*. *coli* has an outer membrane rich in lipopolysaccharides, while *S*. *aureus* has a thicker layer of peptidoglycan. Following either challenge, TEP1_cut_ is delivered to microbial surface by the LRIM1/APL1C complex. TEP1_cut_ is released and covalently bound to the surface. During complement activation by *E*. *coli*, the C-terminal tail is proteolytically processed by an unknown serine protease and SPCLIP1 is recruited and is required for TEP1-F processing activity and CLIPA8 cleavage. During complement activation by *S*. *aureus*, SPCLIP1 is not required for TEP1-F processing but is required for downstream cleavage of CLIPA8.

In this work, we have compared mosquito complement responses to *E*. *coli* and *S*. *aureus* to look for pathogen-specific molecular events. We have made a model summarizing our results (Fig 5). One striking outcome we observed is the proteolytic processing of the LRIM1/APL1C protein complex by a putative serine protease targeting the C-terminus of APL1C. This occurred specifically following *E*. *coli* challenge. LRIM1 and APL1C are Leucine-rich repeat Immune protein (LRIM) family members [11, 26]. The superfamily of LRR-containing proteins has diverse biological functions, but subfamilies of these proteins play important roles in host defense in plants and animals [27]. Prominent examples include the vertebrate Toll-like receptors that transduce a variety of pathogen-associated molecules, and the Variable Lymphocyte Receptors that are the antigen recognition receptors of the adaptive immune system in jawless vertebrates [28]. Two models for the function of LRIM1/APL1C are supported by our data. First, through its LRR domains, the LRIM1/APL1C complex functions as a pathogen recognition molecule. In this model, pathogen binding would induce a conformational change in LRIM1/APL1C leading to activation of a zymogen protease similar to MBL or C1q. In the second model, another pathogen recognition molecule first engages with the *E*. *coli* surface, activating a protease that targets APL1C in the hemolymph LRIM1/APL1C complex. In support of this model, additional putative recognition molecules have been identified by other studies [15, 29–34]. In both models, proteolysis of APL1C could promote the release of TEP1_cut_ near the pathogen surface. The identity of this protease is unknown. Nevertheless, we speculate that the protease activated by *E*. *coli* is important for downstream complement reactions and that *S*. *aureus* either activates a different zymogen protease that does not act on APL1C or that Gram-positive bacteria are directly targeted by TEP1 in an LRIM1/APL1C-independent manner. The latter mechanism is analogous to the alternative pathway for activation of vertebrate complement in that no specific recognition event precedes fixation of a thioester-containing protein. Indeed, overexpression of a refractory allele of TEP1 (TEP1r) that is impaired in the formation of TEP1_cut_ has been shown to be able to participate in killing and melanization of *P*. *berghei* and binding *E*. *coli* [35]. It is likely that individual pathogens may activate different mechanisms of complement activation or that the different systems can work together in a coordinated manner similar to the hierarchical activation of the vertebrate alternative pathway downstream of the classical and lectin pathway activation [25].

Given that, under the current paradigm, the C-terminus of the LRIM1/APL1C complex is required to stabilize TEP1_cut_ [10, 11, 36], it is interesting to consider that proteolysis in this region might promote the release of TEP1_cut_, allowing it to bind the microbial surface and recruit other pathway components, like SPCLIP1. In this case, APL1C proteolysis would be analogous to the C3 activation cleavage performed in the initial stages of complement attack. The C-terminus of LRIM1 may undergo a similar, limited, proteolytic processing; however, we cannot currently address this since the available anti-LRIM1 antibodies are directed against internal regions. It is additionally tempting to speculate that cleavage of the APL1C tail releases a peptide that functions as a positive feedback signal generated during complement protein utilization. Normally, complement components are constitutively expressed and secreted into the hemolymph [9–11, 31, 37]. During *Plasmodium* invasion, these protein components are consumed, and transcriptional activation is required to replenish protein levels [38]. Despite evidence that this mechanism is critical for an effective immune response, the identity of the signal is unknown. Peptides generated during complement activation are interesting candidate feedback signals that may function to promote replenishment of pathway components. Whether cleavage of the APL1C tail has a functional role or simply reflects a difference in the assembly of complement components on different pathogen surfaces remains to be determined.

The loss of APL1C signal we observed in conditioned medium following treatment with trypsin was accompanied by a slight change in the mobility of the LRIM1/APL1C complex, something we did not observe in hemolymph samples exhibiting APL1C signal loss. It could be that trypsin releases a larger fragment of APL1C than that produced *in vivo* during *E*. *coli* infection. An alternative interpretation for the loss of APL1C detection following *E*. *coli* challenge is that the epitope is post-translationally modified and can no longer be detected by the antibody. Post-translational modifications such as phosphorylation and polyubiquitination are common for intracellular proteins functioning in innate immunity [39]. Less is known about modifications of extracellular proteins. However, in mammals, hydrolysis of arginine residues to the non-standard amino acid citrulline is reported during innate immune attack of pathogens by neutrophils [40]. This modification has only a minor impact on the molecular weight of the protein but does remove the positive charge of the residue. There are three arginine residues in the C-terminal tail of APL1C. If such a modification was occurring in the C-terminal tail of APL1C it could abrogate recognition of the epitope by our peptide antibody. In this case, we would still predict that the arginine to citrulline modification process requires a serine protease since treatment with a protease inhibitor prevented the loss of APL1C detection.

Injection of killed *S*. *aureus* is strongly lethal while a similar quantity of killed *E*. *coli* is better tolerated. This observation suggests that a common mechanism with deleterious effects occurs following injection of both bacterial species but to a greater degree following *S*. *aureus* challenge and shows that a pathological response can occur independent of bacterial proliferation. To explain the increased mortality, we hypothesize that *S*. *aureus* promotes a greater immune activation, possibly due to the increased amount of cell wall peptidoglycan compared to *E*. *coli*. In support of this hypothesis, strong activation of the Toll immune pathway using RNAi has been shown to dramatically increase mosquito mortality in different mosquito species independent of an infection [41–43]. Though the mechanism is not understood, strong immune activation at the expense of other pathways is thought to impose resource constraints on the mosquito. An alternative hypothesis is that enhanced lethality observed following challenge with killed *S*. *aureus* might arise from a stronger melanization response than elicited by *E*. *coli*. Melanization produces byproducts that are not only toxic to the pathogen, but also potentially to the host [44]. Furthermore, it has been shown that a non-infectious immune modification that promotes strong spontaneous melanization in the hemocoel, activated by silencing of the serine protease inhibitor, SRPN2, significantly decreases mosquito longevity [45, 46]. Given that the depletion of TEP1-F and SPCLIP1, and the cleavage of CLIPA8 were comparable in the two infection models, another possibility is enhanced lethality is driven by a distinct response due to a feature distinct to *S*. *aureus*. This possibility is supported by the differential effect we find regarding APL1C cleavage as well as previously observed differences in genes required for phagocytosis [16].

## Materials and methods

### Vertebrate animal use

All animal studies were performed under Institutional Animal Care and Use Committee approved protocols and in accordance with the guidelines of the Institutional Animal Care and Use Committee of the University of Pennsylvania (IACUC, protocol A3079-01) and the Imperial College Ethical Welfare and Ethical Review Body (AWERB) in strict accordance with the United Kingdom Animals (Scientific Procedures) Act 1986 under UK Home Office protocol license PLL70/7185 awarded in 2010. The procedures are of mild to moderate severity and the numbers of animals used are minimized by incorporation of the most economical protocols. Opportunities for reduction, refinement and replacement of animal experiments are constantly monitored and new protocols are implemented following approval by Institutional Animal Care and Use Committee of the University of Pennsylvania or the Imperial College Ethical Welfare and Ethical Review Body.

### Mosquito maintenance, gene silencing and infection

*An*. *gambiae* strains used in these studies were N’gousso and G3, the latter was obtained through BEI Resources, NIAID, NIH: *Anopheles gambiae*, Strain G3, MRA-112, contributed by Mark Q. Benedict. Maintenance and gene knockdown were described previously [47]. Conditions for synthesis of double stranded GFP, TEP1 and SPCLIP1 RNA have been reported elsewhere [15, 48].

### Generation and purification of LRIM1/APL1C heterodimer antibody

Three T125 cm^2^ plates of Sf9 cells adapted for growth in serum-free medium (Invitrogen) were each co-transfected with 9 μg of *pIEx10-LRIM1*^*HIS*^ and 21 μg of *pIEx10-APL1C*^*HIS*^ using Escort IV transfection reagent (Sigma-Aldrich). Two batches of conditioned medium (180 mL total) were collected over 6 days, 0.45 μm filtered and supplemented with 0.1% Triton X-100. Conditioned medium was affinity purified in batch purified using Ni-NTA resin (Qiagen) producing LRIM1^HIS^/APL1C^HIS^ heterodimer as well as some LRIM1^HIS^ and APL1C^HIS^ monomers and homodimers, which these cells also produce [47]. The purified protein was used to generate a guinea pig polyclonal antibody (Eurogentec).

### Bioparticles challenge and western analysis

Assays using pHrodo labeled *E*. *coli* (K-12 strain) or *S*. *aureus* (Wood strain without protein A) bacterial bioparticles (Invitrogen) in sterile PBS were performed as previously described [12]. Both *E*. *coli* and *S*. *aureus* bioparticles contain the same number of dead bacteria by weight 3x10^8^ cells/mg. Hemolymph was collected directly into non-reducing SDS-PAGE sample buffer from groups of 30–40 mosquitoes 60 and 240 minutes after the challenge and analyzed by reducing and non-reducing western as described previously [11]. Quantitation of the LRIM1/APL1C complex detected on non-reducing blots using the APL1C anti-peptide antibody was performed using Bio-Rad ImageLab software on non-saturated images. The band intensity was normalized to the loading control PPO6 and compared to the control.

### Protease inhibitor treatment

Groups of approximately 70 mosquitoes 3–5 days post-eclosion were intrathoracically injected with 69 nL of aqueous 0.5 M Pefabloc SC (Sigma-Aldrich) or water alone. After 15 a minute incubation at 25°C, half of each treatment group was injected with *E*. *coli* bioparticles, as described above. The other half was injected with PBS. Hemolymph was collected one hour after bioparticle injection from all groups as well as naïve control mosquitoes for western blot analysis. Prolonged treatment with Pefabloc SC is deleterious to mosquitoes, however, the hemolymph was not grossly affected during the time course of the experiment as shown by the normal abundance and migration pattern of complement proteins in the control.

### Limited trypsin proteolysis of cell conditioned media

*An*. *gambiae* Sua4.0 cells were cultured as previously described [49]. Conditioned medium was prepared by allowing an 80% confluent culture to condition serum-free Schneider’s medium for 3 days [11]. A dilution series of purified mass spectrometry grade porcine trypsin (Thermo Scientific) was created by serial dilution in PBS. 5 μL of the trypsin dilutions were added to 20 μL of conditioned medium for a final concentration in the range of 200–0.8 mg/mL. The reaction was incubated at 22°C for 30 minutes. The digestion was terminated by addition of 6.5 μL of 5x SDS-PAGE buffer supplemented with 5 mM EDTA and a protease inhibitor cocktail. Samples were analyzed by western blot following non-reducing SDS PAGE.

### Survival analysis

*An*. *gambiae* G3 mosquitoes were intrathoracically injected with 69 nL of PBS, *E*. *coli*, or *S*. *aureus* bioparticles in PBS as described above. For each treatment group, approximately 40 mosquitoes 3–5 days post-eclosion were used. The mosquitoes were maintained on 10% sucrose and survival monitored daily.

### VectorBase gene identifiers

LRIM1, AGAP006348; APL1C, AGAP007033; TEP1, AGAP010815; SPCLIP1 AGAP028725; CLIPA8, AGAP010731; ApoII/I, AGAP001826; SRPN3, AGAP006910; SRPN2, AGAP006911.
